# Dysentery—Its Treatment

**Published:** 1871-08

**Authors:** W. T. Goldsmith

**Affiliations:** Atlanta, Ga.


### DYSENTERY—ITS TREATMENT.

BY W. T. GOLDSMITH, M. D., ATLANTA, GA.

It is not the object of this paper to present a new treatment for dysentery, but rather to bring before the reader the methods employed by physicians who have recently written upon the subject, that at one view these various methods may be compared and valuable lessons drawn therefrom. In order, therefore, to make this paper as brief and as practical as possible, I will, at once, proceed to notice the treatment employed by late writers upon the subject.

In an epidemic which occurred in 1868, in Virginia, Dr. F. Horner (Medical and Surgical Reporter,) writes: "It invaded nearly every family, and proved fatal to a third of the number attacked, in defiance of all medical treatment." "The disease was marked by loss of appetite, fever, acute griping pains in the abdomen, mucus and bloody stools, pain and great loss of strength." The treatment employed by him at first, when the cases were mild, consisted in confining the patient to bed—rest—aided by carminatives, of paregoric, powdered kino and lime water, unctions of sweet oil, and farinaceous diet. In severer forms, he used leeches, cups and blisters to the abdomen—gave blue pill and opium, and enforced a strict antiphlogistic regimen. In the latter stages, Dovers' powders, sub-nitrate of bismuth and pulvis ferri carbonatis. Suppositories of morphia and enemas of mucilage and laudanum were administered to allay pain.

In a discussion on dysentery, in the College of Physicians and Surgeons of Louisville, Ky.,—the disease then prevailing—Dr. Wible (American Practitioner, 1870,) remarked: "The cases of dysentery now prevailing appear to be attended with more diarrhea than is ordinarily met in the disease. In treatment I employ opiates at first, to control pain and spasmodic action of the bowels, and afterward castor oil, so as thoroughly to act as a purgative. After the action of the oil, then again I gave opium enough to completely quiet the bowels. Beyond these remedies I give nothing except a sulphuric acid drink, composed of thirty drops of aromatic sulphuric acid, half an ounce of syrup, and half a glass of water. The choleraic tendency of the disease suggested to me the use of the acid, and so far it has appeared to complete the treatment satisfactorily. In the cases treated recently I have given quinine, as there seemed to be a malarious feature in them. In disease of the mucous coat of the intestines, quinine and other remedies fail to produce their usual effects, because they are not absorbed into the circulation. In such the hypodermic method is of precious value; four or five grains of neutral sulphate of quinine injected beneath the skin will be sufficient to arrest a paroxysm, and are equal, I believe, to twenty-five grains administered by the mouth. Opiates may be administered in this way when their effects are not duly produced by the usual method.

Dr. D. W. Yandell, in giving the history of one case, said: "We had tried the salines, opium, quinine, the acids, and astringents without avail. He was growing daily worse. His life was an important one, and we were gloomy lest it should be lost. In the midst of our anxiety Reynolds' System of Medicine reached me from London. I turned at once to the article on dysentery. I was struck by the fact that the author recommended but one remedy for the affection, and that was ipecac. I went myself in the night to the drug-store and prepared the medicine as directed in Reynolds, and administered it to our patient. It acted like magic. Every distressing symptom was at once relieved. The recovery was tedious, because of the exhausted condition of the patient, but it was steady and complete.

I had the honor to read to this College a report of that case along with some others treated at that time. From that time until now I have rarely used in dysentery any other remedy than ipecac. I have almost uniformly trusted to it, and to it alone; and when I have done otherwise I have generally had to regret it. No doubt many cases of sporadic dysentery would recover without it, just as many cases, excited by accidental causes, get well without any drugs whatever; just as the malarial form of the disease yields usually to full doses of quinine; just as a timely dose of oil, followed by a large opiate, will frequently relieve the affection when it depends upon crude ingesta; but as a remedy, in the proper sense of that term, in severe dysentery, in that variety of the affection which, under other management is so often rebellious to the last degree, I know of no agent which approaches ipecac. In my hands it has proved itself all that Dr. Ewart claims for it: "It produces all the good effects that have been ascribed to blood-letting without robbing the system of one drop of blood; of mercurial and other purgatives without their irritating action; of antimonials and sudorifics without their uncertainty; and of opium without masking the disease." I fully agree with Dr. McLean that it is "the most simple, the most successful, the most conservative, and the least distressing mode" of treating dysentery with which I am acquainted. I have used it now in upward of fifty cases with prompt and uniformly good results.

Some months ago a young Texan had a furious attack of dysentery in Galveston. He lay in bed a number of days. He got much medicine, grew better, and started North. At New Orleans he had some premonitions of a return of his disease. When he reached here he was ill. He had tormina, tenesmus, and sixty bloody dejections in twenty-four hours. He had fever, was flighty and without appetite: he was consumed with thirst. He expressed himself so averse to ipecac that for twenty-four hours I trusted to salines and opium. They gave him no comfort. I then administered the ipecac. In six hours his actions changed; his distress was gone. In twelve hours more he was convalescent.

My mode of giving ipecac in dysentery is as follows: Both food and fluids of all kinds are to be withheld for three hours. Give then a hypodermic injection containing a half grain of morphia. As soon as this is done cover the entire abdomen with a light flaxseed poultice, applied very hot, and covered liberally with mustard. Fifteen minutes after give thirty grains of ipecac, rubbed up in just enough peppermint-water to reduce it to the consistency of cream; or, should the patient prefer it, give it as a bolus. Enjoin perfect quiet. Allow a small bit of ice to be dissolved in the mouth, if the patient be clamorous for drink; otherwise interdict all fluids for three hours more. This is the distressing part of the treatment, where the thirst is great. Six hours of abstinence from drink, while in a scorching fever, is a heavy draft upon endurance, one to which few patients submit unmurmuringly. Yet it is most important, if not absolutely essential, in order to prevent vomiting. Sometimes in spite of every precaution nausea and vomiting will occur; but very often, under these circumstances enough of the drug passes downward to produce the thin, copious and brownish dejections characteristic of the action of the ipecac. I have never seen the vomiting unmanageable. It is not apt to occur for two hours after the medicine has been taken, and if the proper precautions are observed it is not likely to occur at all. I generally repeat the medicine in a dose of twenty grs., sometimes only fifteen, within eight or ten hours, and continue it, according to the urgency of the symptoms, at intervals of from twelve to eighteen hours, for two or three days after tenesmus and bloody stools have disappeared. Mild nourishment, milk in some shape being the best, should be given between times."

Speaking of his former mode of treatment, he remarks:

"In an epidemic of dysentery, which preceded and followed a slight epidemic of cholera in this city, in 1850, I thought that the acid treatment then so much in vogue in London, and the treatment by pyroligneous acid, was the best. It certainly seemed very effective—and so for a few years after. But when the "medical constitution" imparted by the cholera-poison had disappeared, the efficiency of the acid and the wood-naptha seemed to have gone too. In 1865, when cholera had made another cycle, I found the acids again most efficient. I gave them at the Dispensary with excellent results. I have not found them particularly useful since, save in exceptional cases. I do not believe that, if that eminent practitioner Dr. Austin Flint were living in Louisville today, he would find opium so useful in dysentery as he found it in an epidemic of this disease which he encountered here fifteen years ago."

Dr. Polk uses the supertartrate of potash and opiates, and gives quinine in almost every case with advantage.

Dr. Wible thinks "the best plan is, first, to completely allay pain and all spasmodic action of the bowels by opiates, and then give the saline purgative until its first effects are produced; and finally, after the action of the salines, to give again the opiates, so as to allay alvine irritation. Such a practice, according to my experience, would cut short nearly any case of the disease."

Dr. James E. Reeves in Medical Notes (Medical Times, June 1, 1871,) writes: "The calomel and opium plan—a cathartic dose of calomel in the beginning, and subsequently one or two grains of this medicine, with opium and ipecacuanha, administered every two or three hours—embracing also, very frequently, general blood-letting, was begun with; and even at the present day (bleeding from the arm perhaps omitted,) this old method of practice has many confident advocates, who, when they are asked for the reason of their faith, adduce experience!

The next plan generally employed was—first, a full dose of castor-oil, and then opium and calomel in small doses administered several times a day, with the view, among other things, of producing ptyalism as speedily as possible.

Another plan was—first, the thorough cleaning out of the primæ viæ by a dose of sulphate of magnesia dissolved in an infusion of peach-leaves—this dose to be repeated every morning, and a full dose of opium or Dover's powder at night—and a diet of soup made of parched wheaten flour. This method of treatment was successfully employed in the Randolph Valley by Dr. Bosworth, of Beverly, who is now one of the oldest physicians in the State.

Another plan was the almost exclusive use of a saturated aqueous solution of the sulphate of magnesia in connection with dilute sulphuric acid, in the proportion of seven ounces of the saturated solution of the sulphate of magnesia to one ounce of the diluted acid—the formula of Dr. Henry, of Dublin—of which mixture a tablespoonful was given every hour until feculent discharges were produced; and this process was repeated every morning until convalesence was established. That it also was successful in a large number of cases, there can be no doubt. The late Dr. D. B. Dorsey, formerly of Ohio, was eminently successful in the treatment of the disease by this mode of practice; and the same plan has been pursued for many years by his son, Dr. D. B. Dorsey, Jr., recently of Fairmont, West Virginia, but now of Chillicothe, Missouri, with equally gratifying results. From the latter I have received a letter giving, in addition to some general directions suggested by their experience in the use of the remedy, the proportions employed by his father and himself, which are as follows:

Saturated solution of Sulphate of Magnesia, f ℥ viij.
Aromatic Sulphuric Acid, f ℥ i.

In his letter Dr. Dorsey says, "Sometimes, after adding the elixir, the mixture solidifies. This is owing to an excess of sulphate of magnesia not dissolved, but *suspended* in the water. The remedy is, therefore, the addition of a little water. I always prefer soft water.

"The dose is from one to three tablespoonfuls, given every 3

or four hours *until fecal evacuations are produced.* This is, in fact, the measure, and the only measure, in administering the preparation. It should be increased in quantity, and even in frequency of administration, until the fecal evacuations appear.—Then the dose should be diminished at once to about one-third of the quantity previously required, and should be continued a day or two.

"While this is in use, no other remedy should be employed, except an opiate at night; as experience has shown that the ordinary routine—astringents, mercurials, etc.—only impedes the proper action of the magnesia. Of course the usual observance of exceedingly rigid regimen should not be omitted.

"Those who have best known and most used this mixture have relied on it *exclusively* in the treatment of simple dysentery; and it has never failed them in a single instance, to my knowledge."

Another plan was the strict prohibition of cathartic medicine after the exhibition of a simple dose of castor oil, to which, usually, ten or twenty drops of laudanum were added to restrain or prevent its violent action, entire reliance being placed on large doses of opium and ipecacuanha. This method of practice gave much encouragement, and found a large number of advocates.

The following is the plan of treatment which I have myself been in the habit of employing during the last ten years in the management of dysentery, and which, indeed, has given me so much satisfaction that I could not be induced to exchange it for any other with which I am acquainted:

1. If constipation have preceded the attack, and the dejections are scybalous in character, a dose of castor oil, with or without ten drops of laudanum, is administered.

II. If preceded by diarrhœa, neither cathartics nor laxatives are to be administered, but the following powder is given in a teaspoonful of the tincture of cinnamon every four or six hours:

℞—Bismuth, subnit., gr. xvj.–xxx;
Cretæ præparatæ, gr. x–xv;
Pulv. Ipecac, comp., gr. iij–vj. M.

One-half to one grain of powdered opium may be either added to or substituted for the Dover's powder.

III. To diminish febrile heat, and control the pulse, the solution of acetate of ammonia in tablespoonful doses, with or without the addition of from one to four drops of Norwood's tincture of veratrum viride, may be given every three or four hours.

IV. Cold water may be allowed as a drink, but should be taken only in small quantities at a time. The diet should consist of boiled milk and bread.

V. To relieve straining and griping, if the bismuth powders fail to secure relief, suppositories containing two grains of acetate of lead and half a grain of acetate of morphia should be employed. For the relief of stranguary, first inject a small quantity of cold water into the rectum, and then introduce the suppository.

VI. Rest in bed is to be strictly observed: no attempt should be made, from first to last, to sit during an effort at stool or when emptying the bladder; and herein lies the secret of success in treatment. In a word, posture is everything in the successful management of dysentery."

Dr. James Henderson, Medical Officer to the Chinese Missiion Hospital, Shanghai, writes that he "found cholera and disentery prevailing to a great extent and proving very fatal. Soon after the middle of September, I became much struck with the rather unusual type which dysentery in most cases assumed A few days after the disease commenced, typhoid symtoms usually appeared, great prostration supervened, frequent pulse with a dry, brown tongue, and sometimes slight delirium. The whole nervous system seemed prostrated, plainly indicating stimulant treatment, and yet the ordinary stimulants produced no beneficial effect. Wine, brandy, ammonia, camphor, mercury, and opium, in many cases seemed useless, either to modify the bowel complaint, or rouse the nervous system; indeed, mercury and ammonia seemed to do mischief. Quinine when retained, appeared to do good, but only in some cases would it remain on the stomach; the mortality was great, and decomposition of the body unusually rapid. On examining the blood of some patients, and comparing it with blood taken from a healthy individual, the former gave a much more decided alkaline reaction than the latter. Even the urine of the patients with typhoid symptoms gave in many cases an alkaline reaction, thus indicating a condition of super-alkaline in the body, or at least a deficiency of acid. This led me to adopt an acid mode of treatment, which proved decidedly beneficial and successful; and as hydrochloric acid enters so largely into the composition of the tissues of the body, I preferred it to the other acids. It is best to give it with some bitter tonic and a little laudanum, if necessary, every three or four hours, and after the second or third dose, a change for the better is in most cases visible. After adopting this method of treatment at the end of September, very few died, if the treatment was commenced before complete prostration of the nervous system ensued. In the treatment of typhoid fever they seem to me as decidedly beneficial and *specific*, as the effects of quinine in ague. Through the "*London Medical Times and Gazette*," I have recommened a *full* trial of this remedy in typhoid fever and in dysentery with typhoid symptoms, to be made in the London hospitals."

Dr. L. Woodruff, Alton, Ohio, recommends common salt and morphine in combination. He says: In September, 1855, I read a single paragraph in Nelson's American *Lancet*, recommending salt and morphia as a remedy in sporadic and epidemic dysentery. Being in the midst of an epidemic at the time, I at once resorted to it, and in every case promptly controlled the bloody and frequent evacuations, and distressing tormina and tenesmus, in from twelve to thirty-six hours. When there are evidences of deranged secretions, I premise the treatment with a dose of calomel, (grs. vj.), and opium (grs. ss.), followed by castor oil, after the operation of which, I give,

℞. Morphiæ, sul., . . . . . gr. j. to iss.

Sodii chloridum, . . . . ℈j.

M. Ft. chart, No. vj.

Sig. Give one every 4 hours.

After the violence of the attack is relieved give at longer intervals. This continued for from one to three or four days, usually completes the cure.

I have suggested the remedy to a number of neighboring practitioners, and invariably received favorable reports.

Of the effects of the prescription, I only "speak that I do know and testify that I have seen."

Dr. Q. C. Smith, Altha, Mo., says: When we are called to a patient presenting unmistakable symtoms of dysentery, we, first, remove all complications, at least as far as time and circumstances will admit of. In other words, our endeavor, so to speak, is to enucleate it from all other diseases. This being done, as far as prudence would indicate, we proceed to administer the following:

℞. Peach-leaf syrup, . . . . . ℥ iv.

Syrup of ipecac, . . . . . . ℥ ij.

Arom. syrup rhubarb, . . . . ℥ j.

Acetate morphia, . . . . gr. viij. M.

Ft. Sol.

S.—Give one teaspoonful every two hours through the day, letting the patient rest undisturbed through the night.

If this amount causes the patient to vomit, diminish the dose until the stomach will retain it.

As to diet, we are not particularly choice, but if the patient can partake of it we direct them to use in the lowest stages, while the stomach is feeble, mutton soup, and the stomach becomes stronger mutton itself, in moderate quantities.

The "Peach Syrup" referred to in the prescription is not officinal, but may be prepared by any one, thus:

Fill a kettle, of any size, with fresh, well-matured, peach leaves, add enough water to cover the leaves, weighting the leaves down, boil gently one hour, remove the leaves, strain the liquor, and while hot add sugar to make a very sweet syrup, and when nearly cool add one ounce of peach brandy. and 2 grs. gum-camphor to each quart of syrup. Let remain in bulk twenty-four hours, then draw off and bottle for use."

Dr. J. F. Kennedy advocates the value of calomel, which he gives in doses varying from 8 to 24 grains, according to the age of the patient, at bed time. He gives a number of cases, (Medical and Surgical Reporter,) and concludes by saying: "I might here give in detail quite a number of other cases, in which the calomel, when administered in large doses, did good service—all recovering rapidly, except one child of three years of age, that died within 12 hours after giving this drug—not because it was given, but in spite of it.

I have not used, nor do I recommend its use indiscriminately. I have found camphor, ipecac, hyd. c. creta, and oleaginous mixtures; a combination of glycerine, nux vomica and carbolic acid; hyoscyamus, and counter-irritants and enemata generally have the desired effect; but in those cases where these seemed inert, and where a sedative was indicated, I have used large doses of the calomel with the greatest satisfaction.

The remedy is not a new one, nor the dose, though it has become very unfashionable in these days of small doses and large "expectancy." It, therefore, requires considerable moral courage to use it or advocate its use, especially in some localities."

Dr. Wm. M. Connell, in speaking of an epidemic of dysentery, (Buffalo Medical Journal,) says:

"All the medicines usually employed in dysentery—castor oil and laudanum, opium and ipecac, acetate of lead, kino, sulphate of magnesia, sulphate of soda, tartrate of potassa and soda, comp. powder of jalap, and the whole range of diaphoretics, wild strawberry, blackberry, etc., all were tried, but apparently did no good. The physician of the village said he did not wish to be called to a case, for they all died.

In this state of things, I was led to look around for some other medicine, and turning over all the books of my medical library—not a very small one—I hit upon the following passage in the first volume of John Armstrong's works, of London, p. 419: "A friend of mine, Mr. George Vaut, of Ipswich, has tried a remedy in dysentery for sixteen years, in about two hundred cases, and the result has been successful, and so remarkably uniform, that I feel it my duty to mention the treatment here. (This was to his medical class.) This gentleman gives in dysentery, or inflammation of the mucous membrane about the colon, seven grains of nux vomica thrice, daily. It neither purges nor constipates, but removes the inflammation, and healthy evacuations follow. Mr. Vaut, who resides in London, bears similar testimony to the value of this remedy, and I strongly recommend it to your notice. I shall certainly try it in the next case I meet with. It seems to operate as a sort of specific. It was first mentioned by Hagstrem, and has been very much neglected since his day."

Upon reading the above, I immediately determined, under the circumstances above stated, to make trial of the nux. I did so, and gave it in the full dose of seven grains, thrice, daily, to adults, and from one to four grains to children, in proportion to their ages. The result was most happy. Not a patient who was treated with this medicine died. It was prescribed in ten cases, within three or four weeks, and all recovered. No cathartic medicine was given, except teaspoonful doses of bitartrate of potassa in a few cases.

It would be presumption to say that this medicine was a perfect specific for dysentery in all cases. Indeed, I am far from having much confidence in the use of specifics generally; but, I am constrained to say, that the above named medicine altogether exceeded my expectations, and I earnestly recommend a trial of it in dysentery.

I had one case of which I almost despaired before using the nux. But the patient recovered under its use. I hope the profession will give this medicine a fair trial. I tried the strychnine, but it did not succeed so well as the nux."

PROF. N. S. DAVIS, Chicago, Ill., seems, however, to have recently (*Chicago Med. Examiner*, *August* 1871,) derived considerable advantage from the use of Strychnia in this disease. In giving the history of one case, he says: "Being satisfied that there was a strong typho-malarial influence in all the cases I had seen in that section of the city, causing great depression in the ganglionic and vaso-motor nervous function, and obtaining no decided effects from quinine, opium, and antiseptics, we determined to try the effects of strychnine and ipecac. We directed twenty grains of ipecac to be give at once, and followed by a teaspoonful of the following solution every three hours, in sweetened water:

| | | | |
|---|---|---|---|
| ℞. | Strychnia, | 1 gr. | |
| | Nitric Acid, | ʒ j. | |
| | Tinct. Opii, | ℥ j. | |
| | Simple Syrup, | ℥ j. | |
| | Water, | ℥ ij. | M. |

At the same time my attention was called to a daughter of the patient, a girl aged about 16 years, who had been taking care of the mother, and who, during the last three or four hours, had felt frequent griping in the abdomen, some nausea, great weakness, and had three or four intestinal evacuations. Her pulse was quick and weak, skin cool and moist, and her expression dejected and anxious. She was directed to keep quiet and take one teaspoonful of the strychnine mixture directed for the mother, every four hours.

At my visit the next day, the girl was entirely comfortable, having had but one passage after she commenced taking the medicine. She took it three times a day for two days longer, and remained well.

The mother was also much better. The dose of ipecac was followed in a few minutes by free vomiting, but she retained the strychnine solution, and her intestinal discharges had been diminishing in frequency, until they do not now occur more than once in three or four hours, and contain very little blood. The pulse, the capillary circulation in the surface, and the temperature have also much improved. She was directed to continue the strychnine solution in the same doses every four hours, and limit herself to wheat flour and milk porridge for nourishment, which she did for two days more, when all symptoms of dysentery and fever had disappeared. She continued the medicine three times a days longer, during which time she cautiously returned to an ordinary diet, and her recovery was complete."

Dr. W. T. Gairdner, highly recommends creosote in acute dysentery. He has given as large a quantity as one drachm at a dose.

Prof. N. S. Davis, uses carbolic acid, under the following circumstances: "To allay the irritability of the stomach and correct in some measure the offensive quality of the intestinal discharges, we ordered the following mixture to be taken in doses of one teaspoonful every two hours:

| | | |
|---|---|---|
| ℞. Carbolic Acid Crystals, . . . . | 6 grs. | |
| Glycerince, . . . . . . . | ℥ ss. | |
| Camph. Tinct. Opii, . . . . . | ℥ jss. | |
| Water, . . . . . . . | ℥ ij. | M. |

Also one of the following powders every four hours:

| | | |
|---|---|---|
| ℞. Tannate of Quinine, . . . . . | 30 grs. | |
| Pulv. Opii, . . . . . . | 10 grs. | M. |

Divide in six powders.

Dr. Ragland advocates veratrum viride (The Medical Archives,) in dysentery, and alludes to a case of a boy, æt. six, who, when first seen, had from thirty to forty evacuations in twenty-four hours; pulse rapid, small, and wiry, numbering 130 per minute, and the temperature, as indicated by the thermometer in the axilla, 103.5° F. Wishing to reduce the too rapid action of the heart, he gave veratum viride, commencing with three drops Norwood's tincture, and increasing one drop each dose, which, in six hours, brought the pulse from 135 to 60 or 70 beats, and in corresponding ratio the temperature was found to have fallen to 101° F.

The marked benefit of the treatment was shortly manifested in the lessened number of evacuations and reappearance in them of fecal matter. The child made a good recovery, convalescing steadily from the time of the favorable change.

Experience induces Dr. Gordon (Medical press and Circular,) to believe that in cases not only of inflammatory dysentery, but in those of the hemorrhagic type of the disease, enemata of hot water repeatedly administered are not only to be highly recommended as means of treatment, but that they afford an amount of relief to the patient that by no other means can be obtained. Dr. Gordon does not mean by this the repeated introduction of long enema tubes, such as were some years ago in common use in India, but the administration of lavements in the ordinary manner. Nor can he too strongly urge the great benefit and relief derivable from the very simple means of permitting patients affected with dystery to sit upon commodes containing hot water. The vapor soothes the pain of straining in a manner that nothing else does, and thus no doubt becomes a powerful means of cure. Dr. Gordon recommends in cases of hemorrhagic dysentery, that, where gallic acid fails to check the flux, a solution of alum with diluted sulphuric acid added, or of acetate of lead with acetic acid, should be tried.

Dr. C. T. Hart thinks the Dioscorea Vilosa (Wild Yam) a fine remedy, in combination with others, in dysentery, giving prompt relief in tormina and painful tenesmus. It is particularly useful in those cases which do not bear opiates well.

Ergotin has been found very useful. [*Half-Yearly Compendium of Medical Sciences.*] Gros (*Algem Weiner Med. Ztg.*, No. 25, 1868, and *Allg. Med. Cent. Ztg.*, No. 58, 1868), recommends this remedy.

He treated 44 cases of dysentery with it, and had but one death, this being caused by dietetic irregularity on the part of the patient; 25 of the cases were of a mild type.

He gives 6 grains in mucilaginous emulsion; and frequently also gives clysters of 12 grains, in starch.

Where it is not desirable to check the discharges too suddenly, as in acute diarrhœa, the enemata alone are used. He also speaks favorably of its effects in the chronic diarrhœa of adults and children; the latter take the remedy best in the form of *dragees.*

The clysters are particularly useful as hæmostatics in the bloody dejections of dysentery.

In one case two doses and two clysters were given,—the latter, two days subsequent to the former; cure followed within six days; but there was a slow convalescence owing to unpropitious circumstances.

At the Rudolf Hospital, Vienna, the Chlorate of Potash has been used by enema, with excellent results—blood ceased to appear in the dejections after the first clyster. They used potass chlor., ℈i ad. aqua distil. ℥ij.

Injection of nitrate of silver have been administered with good success, by the following plan, viz: Crystalized nitrate of silver, 25 grammes; distilled water, 200 grammes; to be used in the following manner: After each injection of the solution, another injection of about 300 grammes of tepid water immediately to be given, in order that the nitrate may be conveyed high up into the intestine, and come in contact with a larger extent of surface.

Dr. Thomas T. Gallaher, in a valuable article furnished the New York Medical Journal on Hypodermic Injections, remarks:

"During last summer I treated three cases of dysentery by hypodermic injection of morphia with complete success. I append the three following cases, one from notes taken at the bedside, the others from memory, as illustrative of the treatment.

These cases show that not only the pain and tenesmus of dysentery may be instantly relieved by the hypodermic injection of morphia, but the disease itself may be entirely cured without the employment of any other remedy. The cure, too, is much quicker than by the usual method, and the administration of frequent doses of nauseous drugs obviated. From one to two injections, mostly but one, daily, is all that is required. I have resorted to this method also in semi-chronic forms of dysentery and diarrhœa with entire success.

It is probable that a combination of Ergotin and Morphia would give good results in this disease, used hypodermically.

Dr. Frazer's (of St. Louis,) method of treating chronic dysentery is well worth the space we afford it. He says:

I commence the treatment by giving two large tablespoonfuls of the decoction of simaruba every four hours, and one large tablespoonful of Hope's mixture between each dose of the simaruba, or, in other words, I give the two preparations named above in the doses named, every two hours—taking the same medicine only every four hours. During the administration of these remedies, and for some weeks after their discontinuance, I give an infusion of frostwort—a medium sized wineglassful of the t. a every two or three hours. I prepare the tea by adding one quart of boiling water to one ounce of the frostwort.

I persevere in the above treatment until the bowels are corrected, when, if the patient be anæmic, as usually happens in such cases, I give, in connection with the frostwort tea, the syr. proto. nitrate of iron, twenty drops three or four times a day for two or three weeks.

During the progress of the case the patient should be restricted to a milk diet; indeed, in the more aggravated cases, I do not permit them to use any other diet whatever, not even a crumb of bread. I deem it of the utmost importance that the bowels remain perfectly at rest.

Below I give the recipes as prepared for me by Mr. Eugene S. Massot, druggist, at corner of Spruce and Fourth streets. I consider it of the greatest importance, in the treatment of the above disease, that the medicines should be fresh and pure, and prepared with the greatest care and circumspection.

The following is my formula for making the Compound Infusion of Simaruba:

| | | |
|---|---|---|
| ℞. | Simaruba Bark (bruised) . . . . | ʒ vj. |
| | Boiling water, q. s. to make ℥ 12 of the infusion; strain and add Holland Gin, . . | ℥ iv. |
| | Loaf Sugar, | ℥ ij. |

Bottle for use.

I also append the following account of the celebrated Hope's nitrous acid mixture, taken from the *Edinburgh Medical and Surgical Journal*, 1824, entitled, "*Observations on the powerful effects of a mixture containing Nitrous Acid and Opium in curing Dysentery, Cholora and Diarrhœa, by Thomas Hope, Esq., Surgeon, Chatham:*" More than 26 years ago, when attending a case of dysentery, in which the usual remedies had been prescribed in vain, the patient determined on his own accord to take a medicine I had sent for his nurse, who was worn out with attention to her charge, and complained of excessive thirst. It occurred to me to give an acid to alleviate her complaint, and in order to obviate any unpleasant effects to join opium with it. I accordingly sent the following:

| | | |
|---|---|---|
| ℞. | Acid Nitrosi, . . . . . . | ʒ ii. |
| | Ext. Opii, . . . . . . | grs. ij. |
| | Aqua, . . . . . . . | ʒ ij. |

Cap. Cochl minus ter quarterve in die. M

And the patient with dysentery having taken some of this medicine, the effect produced was so great that it no less surprised him, who, by a continuance of it, recovered, than it did myself

The form of the medicine, as I used it in all the cases referred to, is as follows :

℞. Acid Nitrosi, . . . . . . ʒ i.

Mist. Camphor, . . . . . ℥ viij.

Misce et adde

Tinct. Opii, . . . . . . gtt xl.

Sig : One fourth part to be taken every three or four hours.

In chronic dysentery the dose of two ounces, three times a day is quite sufficient. The remedy is grateful to the taste, abates thirst, soon removes the intensity of pain, and procures, in general a speedy and permanent relief. No previous preparation is required for taking it, nor any other care whilst taking it, except the keeping of the hands and feet warm, preserving the body as much as possible from exposure to extreme cold or currents of air, and making use of warm barley-water or thin gruel and a diet of sago or tapioca. It is necessary to mention that the remedy, the good effects of which I now detail, is nitrous acid, with opium—*not* nitric acid, with opium. I have *not* found nitric acid, with opium, to produce any good effect; for having expended my nitrous acid, I sent to a chemist for a fresh supply, who, by mistake, sent me nitric acid, which I used merely by way of trial, but I found it not in any way beneficial to my patients.

In conclusion, I can say to my professional brethren, that no treatment by me or under my observation, approximates the success of this in all the purely chronic cases of dysentery and diarrhœa."

Dr. E. M. Morse recommends the following treatment for chronic dysentery :

In chronic simple uncomplicated dysentery, by which are meant those cases not kept up by organic disease of the heart, or phthisis pulmonalis, nor dependent on irremediable obstruction of the liver or spleen, Dr. Morse has met (California Medical Gazette,) with marked success by throwing up into the rectum and colon from two to five pints of Labarraque's solution of chlorinated soda, largely diluted. The theory of the treatment is based on rational principles, and the remedy gives little or no pain, while experience has demonstrated that it is perfectly safe, no bad effects thus far having been observed. Dr. Morse says :

"In order to get the patient into a proper condition to derive the most benefit from these injections, I am in the habit of pursuing the following method : I regulate his diet carefully, of course, and keep him in a recumbent position in order to assist the return of the blood from the engorged mesenteric veins, and those smaller tributaries which are distributed along the large intestine. This state of engorgement prevents the ulcers from healing, and renders each ulcer an outlet from which, in blood and serum, the stream of life ebbs out like water from the tubs of the daughters of Danaus. At daybreak on every alternate or fourth day I give a mild cathartic or aperient, in order to clear out the alimentary canal. The ordinary contents of the intestine produce great irritation when it is in this engorged and hyperæsthetic condition; and it is better to get rid of the feces about the same time instead of letting them run in driblets over the raw surface every hour or two. After the cathartic or aperient has acted sufficiently, I inject very slowly from two to four pints of Labarraqu's solution of chloric of soda, diluted, into the large intestine. In this way it becomes a topical application. The right strenth for the first enema is twenty parts of water to one of Labarraque's solution. I inject as much of this mixture as he can be made to retain. Two or three pints will generaly be enough. Sometimes as much as five pints may be given. Each enema should be made a little stronger, until the patient says that he can feel it smart or burn. When this happens the solution is of the proper strength. The patient should be on his right side, or on his knees with his head low down, while these enemas are being administered. Occasionally it is necessary for him to change his position several times in order that the wash may reach every point where it is needed. Should there be much tenesmus after the injection has been passed, I give an enema of the tinct. opii, or an opium suppository. These applications of the chloride of soda should generally be made once a day. Occasionally it is necessary to give them twice a day, and sometimes, on account of this sensitiveness of the ulcers as they begin to heal, it is better to leave them off for several days, or give weaker solutions. The next indication in the treatment, after cleaning out the alimentary canal and washing the ulcers with the medicated solution, is to keep the bowels quiet, so that the ulcers may remain clean and heal up under the topical application. In suggesting the means of accomplishing this desideratum I am getting upon very debatable ground. The old proverb, '*tot homines, tot sententiones*,' must certainly have been intended for physicians. Each one of us has his own way of using the arms with which we combat disease. I generally give large doses of subnitrate of bishmuth three times a day; repeated opiate enemas and suppositories, in order not to disorder the stomach; Dover's powders, repeated if necessary; charcoal, or the mineral and vegetable astringents; the antacids, leeches, and fomentations; taking great care to keep up the effect of the medicines by giving them every hour or two. If one drug fails I try another, or give a combination of several of them, in order to have as few stools as possible passing over the ulcerated surface while they are healing."

---

Tincture of Calendula in Phagedæna.—Thomas Kennard, of St. Louis, Mo., (Med. Archives.) advocates the use of the tincture of calendula (garden marigold,) as a local application in phagedæna. He has used it in preference to aromatic wine as a dressing for syphilitic sores for many years. It is also recommended as a stimulant and healing application for lacerated wounds, ulcers, or any breach of continuity of the surface.

The Therapeutic Value of Gelseminum.—Dr. Philip C. Williams, Baltimore, Md., (Baltimore Medical Journal,) in an extended paper on the value of "Gelseminum," believes that it will cure all pure, simple neuralgias of the cerebral system with promptness and efficiency; that it will relieve cerebral congestion; that it controls maniacal excitement, and a variety of conditions resulting from derangements of the central nervous system. For these and analogous disorders he recommends gelseminum as a most powerful and efficient remedy.

Dr. J. Marion Sims says, (N. Y. Med. Gazette,) "I have had but a limited experience with this new extract of Pinus Canadensis, but I am so well satisfied of its value that I am anxious to call the attention of the profession to it. I have used it for about eight months in some affections of the rectum, vagina and cervix uteri; I have used it, considerably diluted, as a vaginal wash, with great success; but I prefer to apply it to the os tincæ on cotton wool, either pure or mixed with glycerine, or glycerine and rose water. Thus applied, it should remain intact for two or three, or even four days, and then be renewed. In this way I have seen chronic granular vaginitis remedied in a few days that had resisted the ordinary remedies for weeks; and I have seen granular erosions, with leucorrhoea, disappear very rapidly under its use. I have not time to do more than call the attention of my professional brethren to this new extract, which I am sure will soon be recognized as a valuable addition to our Materia Medica."

